# Effects of isolation, crowding, and different psychological countermeasures on crew behavior and performance

**DOI:** 10.3389/fphys.2022.963301

**Published:** 2022-11-15

**Authors:** Dmitry Shved, Polina Kuznetsova, Ivan A. Rozanov, Svetlana A. Lebedeva, Alla Vinokhodova, Alexandra Savinkina, Ksenia Shishenina, Nicole Diaz Rey, Vadim Gushin

**Affiliations:** ^1^ Russian Federation State Scientific Center, Institute of Biomedical Problems of the Russian Academy of Sciences, Moscow, Russia; ^2^ M. V. Lomonosov Moscow State University, Moscow, Russia

**Keywords:** space analogs, ground-based space simulation, isolation, confinement, crowding, mixed-gender crew, ESKIS

## Abstract

Studies conducted by I. Altman in the 1960–70s revealed the increase in the individual stress level under isolation and confinement. Altman introduced the term “privacy” as a desired level of personal space that humans need to feel psychologically comfortable. The author also mentioned the dynamic process of boundary regulation that can be accompanied by the increase in conflict tension in the confined groups. In our study with short-term chamber isolation ESKIS, we analyzed behavior, crew interactions, and psychological state of a mixed-gender crew with none or minimal previous isolation experience (4 males and 2 females) who spent 14 days in a small chamber of 50 m^3^. The study confirmed that the pre-isolation period was particularly stressful for the subjects who felt also significant anxiety during the first days of isolation. Also, some mood and sleep disturbances were detected under isolation and crowding. Psychological stress made the crew more cohesive; they demonstrated the increase in common values. Extraverted subjects who could obtain social support from their partners and Mission Control’s duty teams were less interested in psychological support *via* VR.

## Introduction

Isolation chamber studies conducted by I. Altman (1975) detected the increase in some physiological parameters (heart rate, breath frequency, etc.) that testified to the increase in stress under confinement. He attributed that stress has the ability to penetrate into the individual’s personal space and introduced the term “privacy” as a desired level of personal space that humans need to feel psychologically comfortable. He also mentioned the dynamic process of boundary regulation that can be accompanied by the increase in conflict tension in the confined groups of various cultures.

Further chamber studies in the Soviet Union (Kitaev–Smyk and Bobrova, 1988) confirmed the existence of territorial behavior, when subjects tried to define their own psychological space and keep its boundaries. Every penetration into personal space could cause aggressive behavior. Also, several studies that were conducted in densely populated urban territories (Steinbach and Yelenskij, 2004) showed the increase in psychophysiological stress and conflict tension related to crowding and lack of privacy. The psychophysiological effects caused by the spread of COVID-19 demonstrated the lack of data concerning the impact of long-term isolation and confinement on the individual’s psychological state and small group dynamics, as well as the appropriate countermeasures under these conditions (Allé and Berntsen, 2021).

A number of studies were conducted with mixed-gender groups under extreme conditions of the Antarctic environment, including long-term isolation at research stations (Palinkas et al., 2000; Steinach et al., 2016; Strewe et al., 2019). The isolated and extreme environment of Antarctic stations was considered analogous for space missions, and extensive research on the winterers’ psychological state, behavior, and performance was performed (Palinkas et al., 2000). In recent studies, it was shown that generally, an extreme environment causes distinct stress responses (measured with physiological methods), as well as significant changes in sleep patterns. However, in most cases, no significant impact on the self-reported (questionnaires) psychological well-being of the subjects was revealed in these studies. It may also be challenging to distinguish between the effects of the harsh Antarctic environment (extreme temperatures, lowered atmospheric pressure and oxygenation, solar radiation, etc.) and the ones of isolation and confinement.

Space simulations that are carried out by space agencies seem to be one of the most appropriate analogs to analyze not only the effects of the extended space flights but also the effects of extended stay in isolation and monotony on Earth. The advantage of the chamber studies as one of these analogs is the artificially created environment with controlled parameters as well as the opportunities for complex observation by the objective measurements of several parameters of the subjects’ behavior, interaction, and performance (Gushin, 2021).

To simulate isolation and crowding, the Institute of Biomedical Problems of the Russian Academy of Sciences (RAS) launched the 14-day chamber experiment, ESKIS (abbreviation from “experiment with short-term isolation” in Russian), with a participation of six subjects, of which 5 had no experience in participating in the experiments with isolation and confinement. It has been established previously that the first 2–6 weeks of space flight or an analog experiment is a period of acute adaptation to the extreme (or unusual) living conditions, usually associated with stress development (Kozerenko et al., 2001). According to our first hypothesis, the lack of privacy and personal space combined with the uncertainty of expectations could cause an increase in the anxiety level and conflict tension within the crew. The increasing stress levels could exert detrimental effects on the subjects’ psychophysiological and psychosocial well-being, impairing their mood, cognitive performance, overall activity, sleep quality, and crew cohesion. According to the second hypothesis, we expected that subjects with better developed communicative skills and extroverted personality would obtain psychological support (PS) mostly *via* contacts with the crew and supporting group. On the other hand, subjects with dominating introversion could have problems with obtaining social support to withstand sensory deprivation, monotony, and crowding. For them, PS *via* computerized content, including a virtual reality environment, which is clearly dosed, contains preliminary defined relaxing data, and provides additional virtual space, should be the most preferred type of psychological support.

## Materials and methods

### Participants

A total of six healthy volunteers, four men and two women, aged 23–45 years, all Russian, with no or minimal previous experience in space simulations with isolation, were selected by the IBMP Medical Board.

To preserve the confidentiality of the subjects, each of them is designated by a code (A, B, C, D, E, and F).

### Bioethics and informed consent

The studies involving human participants were reviewed and approved by the Bioethical Commission of the Institute of Biomedical Problems of the Russian Academy of Sciences and fully complied with the principles of the 1964 Declaration of Helsinki.

Each study participant voluntarily signed informed consent after having the potential risks, benefits, and nature of the upcoming study explained to them.

### Design of the study

**FIGURE 1:**
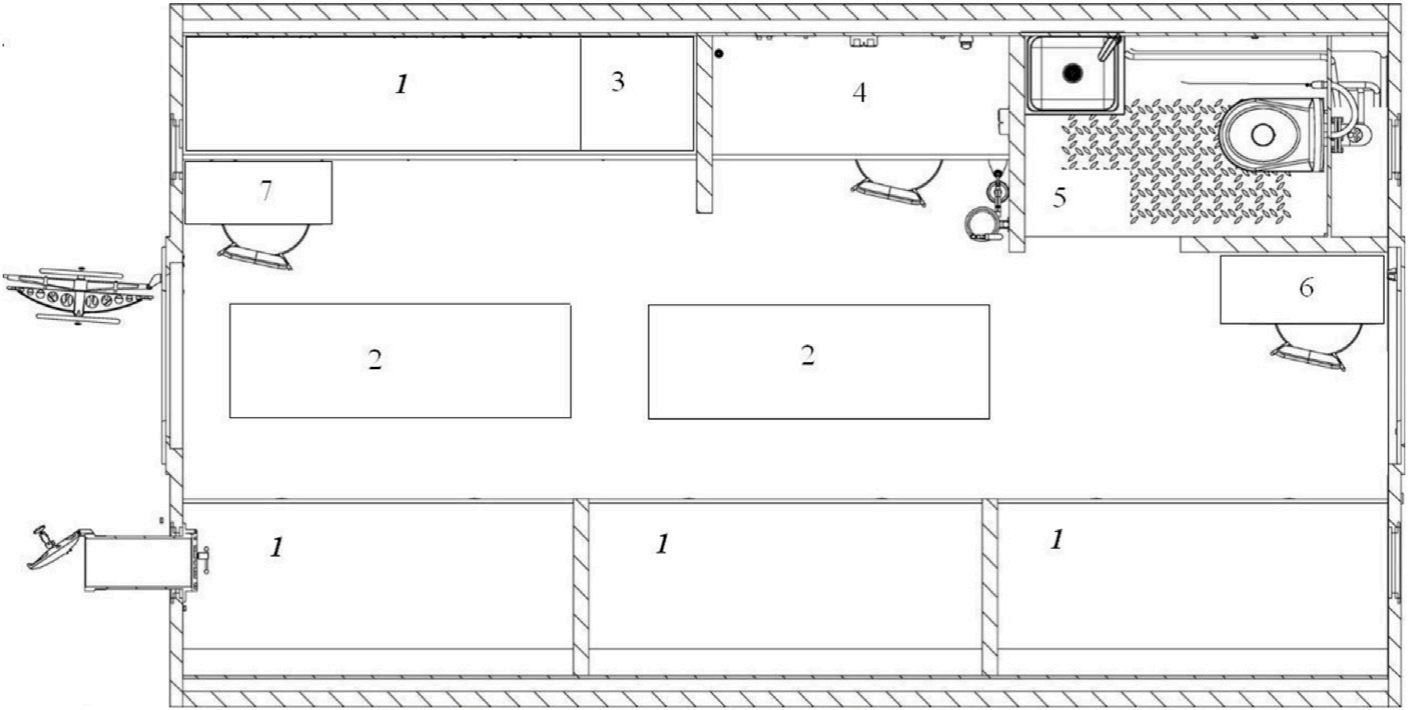
EU-50 chamber floor plan: 1) stationary sleeping places (berths), 2) temporary sleeping places (mattresses), 3) rack with a microwave and kettle, 4) workplace, 5) bathroom/WC (no shower), 6) additional workplace (folding table), and 7) serving table.

The ESKIS (experiment with short-term isolation, which when translated into Russian gives “ЭСКИЗ–Эксперимент С Короткой Изоляцией,” lit. “sketch”) experiment with 14-day isolation and crowding of the mixed-gender crew was carried out in a chamber of 50 cubical meters (18 m^2^ of living space) at the SSC RF-IMBP RAS (Nosenkova, 2021). This habitable volume is comparable to the living space of orbital stations (Salyut-type stations have a volume of 90.0 m^3^, the Destiny ISS module has a volume of 117 m^3^, and the International Habitat Module of the upcoming Lunar Gateway station will have a volume of 125 m^3^). However, spaceships and modules of orbital stations comparable to the habitable volume of the module in the ESKIS experiment should be considered psychologically more spacious, since astronauts can use all available surfaces due to weightlessness. The set of furniture in the ESKIS experiment was strictly limited and included two folding tables for work and eating, folding chairs, and a stationary table with equipment for studies and communication with Mission Control (Figure 1). The number of berths in the module was less than the number of crew members, so two additional sleeping places were arranged in the aisle.

The daily routine was specified in accordance with the experiment program, including execution of the operator’s performance tasks, three meals, 8 h of sleep, a medical supervision program, and experimental studies.

The main psychological factors affecting the subjects during the experiment are typical for chamber confinement studies (as well as isolation caused by epidemiological reasons): sensory deprivation, monotony, crowding, lack of privacy, and the limitation of social contacts (Feichtinger et al., 2012).

## Methods

To estimate subjects’ personality, mood, and behavior, several psychological methods were applied. They included:

1) POMS questionnaire (Profile of Mood States)—a psychological rating scale used to assess mood changes—applied once before and after isolation and four times during isolation (on mission days (MD) 2, 6, 9, and 13). The POMS has been previously used to assess the mood of astronauts as well as subjects in model experiments (e.g., Boyd et al., 2007).
2) Keirsey Temperament Sorter (KTS, with extraversion–introversion, sensation–intuition, thinking–feeling, and judging–perceiving scales) was utilized to estimate the influence of personality traits on the psychological adaptation and preference of psychological support measures—once before isolation.
3) Spielberger’s State-Trait Anxiety Inventory (STAI) was used to monitor the anxiety level. The questionnaire was utilized in a variety of experimental conditions, including extreme ones (e.g., Strewe et al., 2019).

To estimate changes in crew cohesion and group dynamics, we used the following:

1) The value–orientation test proposed by M. Rokeach was applied to estimate cohesion (Rokeach, 1973).
2) The sociometric questionnaire was used to evaluate the sociometric status (“popularity”) of each crew member and group cohesion by counting the number of received sociometric choices in two situations (professional and leisure)—before, after, and three times (MD1, MD6, and MD13) during isolation.
3) PSPA (Personal Self-Perception and Attitudes) (Vinokhodova and Gushin, 2014)—software for analyzing subjects’ attitudes toward a social environment based on a personal construct approach (Bannister and Fransella, 1986). Analyzing the distances between the subjects in their psychosemantic two-dimensional space, we focused on the general trends in the perceived similarity (psychological closeness) or, in contrary, dissimilarity (psychological distance). The crew performed the test before and after the mission and twice (MD6 and MD13) during isolation.

In order to monitor the psychophysiological state and cognitive functions of the subjects, we utilized:

1) Actigraphy to evaluate motor activity under isolation and crowding using constantly worn Garmin Fenix 6X and ActiGraph wGT3X-BT devices.
2) ActiLife software was used to analyze sleep quality based on actigraphy: the time spent in bed, sleep duration, and number of night awakenings were calculated using the Cole–Kripke algorithm (Cole et al., 1992)—during isolation and 7 days before and after it.
3) Morning and evening daily planning conferences (DPCs) containing the subjects’ semi-structured video reports about their mood, health, and performance were analyzed using FaceReader 7.1. software to estimate their facial expressions (Küntzler et al., 2021). Acoustic analysis of the reports using Praat software was also performed, including parameters such as fundamental frequency (F0), signal intensity (speech volume), number of pulses and unvoiced fragments, jitter, and shimmer (Lebedeva & Shved, 2022).
4) Daily sensorimotor and cognitive tests simulating some aspects of operator activity: reaction to a moving object (RMO, extrapolation and coordination tasks), short-term memory tests, simple mathematical calculation, and response speed tests (Ushakov et al., 2015).

To prevent negative effects of staying under sensory and social deprivation, monotony, and confinement, psychological support (PS) measures that are widely used in International Space Station flights were applied (Rozanov et al., 2021). The PS sessions included providing crew members with personal multimedia content (videos, music, and photos), chosen by themselves before isolation, which subjects could access on personal tablets. During VR-based PS sessions, software, which was presented *via* a VR helmet, allowed users to access an additional virtual personal space, an interactive image of a personal recreation room. In this virtual room, the subjects could change the interior, the weather and time of the day outside the virtual window, and the light in the fireplace according to their individual preferences. A set of personal multimedia content was available from this room for listening and viewing. Also, subjects had the opportunity to make drawings as a sort of art therapy. Sessions of each type of PS were conducted four times during isolation for each crew member. The questionnaire “psychological support” allowed to estimate the degree of support from various sources such as multimedia, VR, and communication with the crew.

### Statistical analysis

For statistical analysis, the Wilcoxon *t*-test, as well as the Spearman and Pearson correlation, was used, with consideration of the sample sizes, types and distribution of the variables, and analysis goals. The analysis was carried out using SPSS software.

It should be noted that the small sample size (considering the number of subjects) poses a significant limitation to the present study, although the experimental design allowed frequent data collection for each subject.

## Results

### Psychophysiological status and mood

**FIGURE 2:**
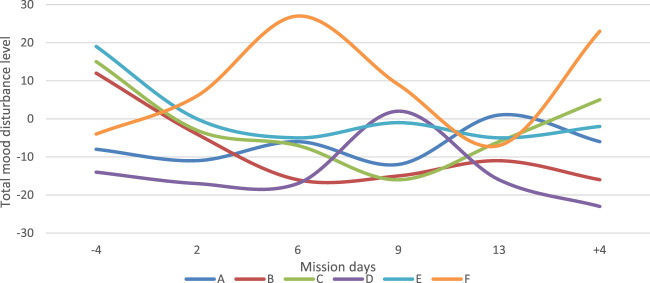
Dynamics of negative changes in the mood (total mood disturbance level) of the participants in the experiment according to the results of the POMS questionnaire.

Analysis of the negative mood states (tension or anxiety, anger or hostility, fatigue or inertia, depression or dejection, and confusion or bewilderment) by applying POMS before, during, and after the isolation period revealed the increase in negative feelings before the chamber study, on the first day of it, and after the isolation period for most of the crew. For two subjects, D and F, some increase in negative emotions was also detected in the middle of the mission. However, in general, no tendencies for the increase in tension, anxiety, and depression were found throughout isolation (Figure 2). More or less, the same results were obtained using the STAI test; a pronounced increase was detected only before and during the first day of isolation. These changes could be attributed to the preparatory stage and acute period of adaptation to the experimental conditions.

**FIGURE 3:**
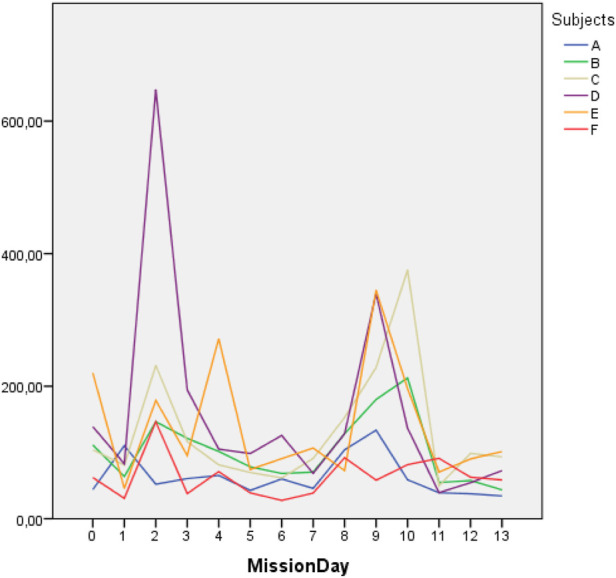
Dynamics of the evening DPC duration (in seconds) in isolation.

The dynamics of the duration of morning and evening DPCs also demonstrated only two major spikes on 2–3 days of isolation and after the simulation of lunar extra-vehicular activity (EVA) when crew members moved in pairs to a bigger chamber and performed for 45 min a number of complex operator’s tasks (Figure 3). In general, the duration of the evening video reports decreased (*p* = 0.043).

The analysis of the expression of basic emotions during DPCs demonstrated that for four subjects, the higher was the duration of the DPC, the lower was the expression of happiness (*p* = 0.023), surprise (*p* < 0.001), valence (*p* < 0.001), and the level of excitement (*p* < 0.001). At the same time, sadness (*p* = 0.019) and anger (*p* = 0.008) increased in this group during the isolation period. For two subjects, no dynamics in emotional expression was found. For subject D, the tendency for growing neutral emotions in the facial expression was found (*p* = 0.015). These data correspond to the previous results from longer isolation studies MARS-500 and SIRIUS-19 (Supolkina et al., 2021).

Some correlations between acoustic parameters, such as the number of pauses, speech loudness, and shimmer effect (variation of the voice amplitude), during DPCs and the STAI anxiety level were revealed. Аnxiety levels were negatively correlated with speech loudness and positively correlated with the number of pauses and shimmer effect (Table 1). Also, we found that the increase in the anxiety level was positively correlated with the number of errors and time for mathematical calculation in cognitive tests.

**TABLE 1:** Results of the correlation analysis (Pearson’s correlation coefficient): acoustic characteristics of speech, cognitive, and sensorimotor performance, situational anxiety, and the number of days of experimental exposure.

		State-Trait Anxiety Inventory (STAI)
Speech signal intensity	Pearson’s correlation	−,391^**^
	Sig. (two-tailed)	,000
	N	101
Percentage of pauses (unvoiced fragments)	Pearson’s correlation	,270^**^
	Sig. (two-tailed)	,006
	N	101
Shimmer	Pearson’s correlation	,359^**^
	Sig. (two-tailed)	,000
	N	101
Time spent on calculations	Pearson’s correlation	,328^**^
	Sig. (two-tailed)	,001
	N	102
Errors in calculations	Pearson’s correlation	,245^*^
	Sig. (two-tailed)	,013
	N	102

### Motor activity and sleep

The study of motor activity under confinement and crowding showed an expected decrease of this parameter. Motor activity in the small 50-m^3^ chamber was 30% lower than that during the preparatory stage and 29% lower than that during the recovery period.

**FIGURE 4:**
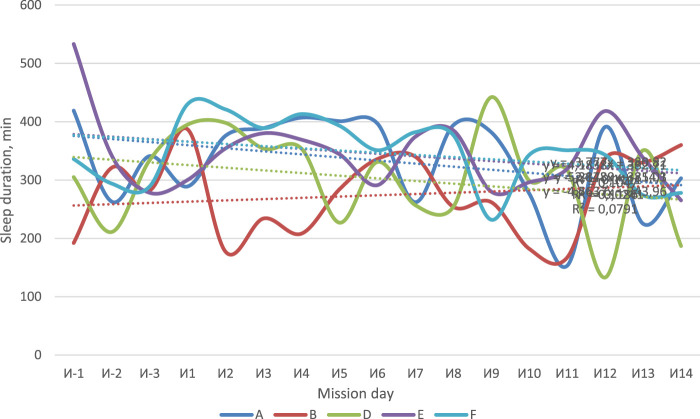
Duration of subjects’ night sleep according to actigraphy.

**FIGURE 5:**
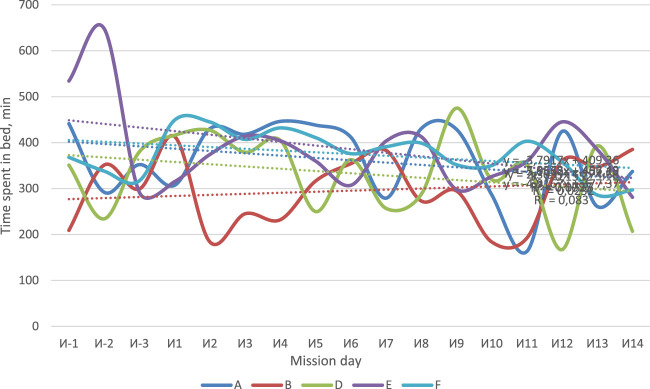
Time spent by subjects in bed, according to actigraphy.

**FIGURE 6:**
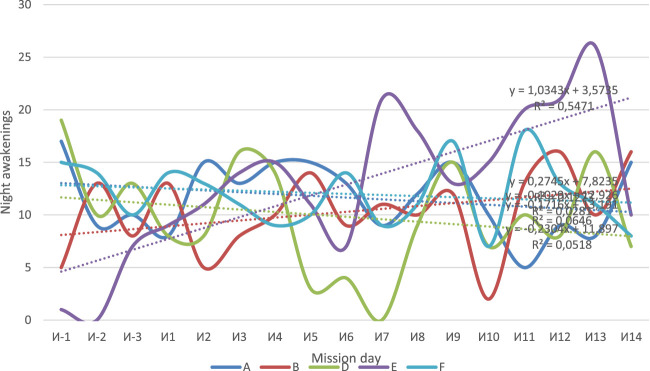
Number of nocturnal awakenings in the subjects according to the actigraphy data.

The use of actigraphy made it possible to assess the duration and quality of sleep (including 3 days of the baseline period and 14 days of isolation). For the whole crew, except B, the decrease in sleep duration was observed (Figure 4). All subjects, except B, had a tendency to decrease the amount of time spent in bed which was defined by analyzing actigraphy data using the Cole–Kripke algorithm (Figure 5). The number of night awakenings for subjects A and D increased in the course of isolation (Figure 6). In general, sleep duration and quality were negatively affected by experimental conditions.

### Group dynamics

**FIGURE 7:**
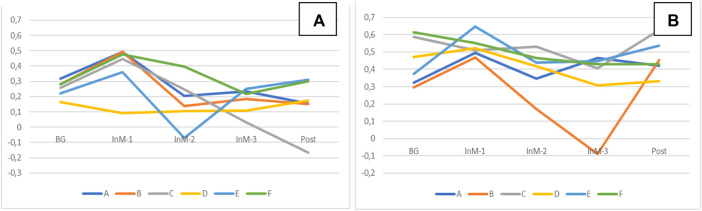
Dynamics of the object–value unity of the group in terms of positive **(A)** and negative **(B)** qualities (arbitrary units).

**FIGURE 8:**
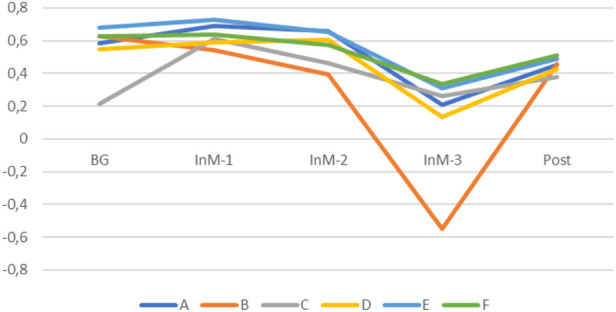
Dynamics of the subject–goal unity of the group (arbitrary units).

Analysis of the value–orientation unity in the group demonstrated that for the positive traits (values), the subjects, in general, showed similar dynamics during isolation, except for two of them (Figure 7A). At the same time, for the values which the group regarded as unacceptable under isolation, subject B demonstrated negative dynamics, while the other subjects became psychologically closer (Figure 7B). Analysis of the common goals (subject–goal unity) produced similar results (Figure 8). In the group, five members became close in the goals to reach during isolation and one of them demonstrated increasing inconsistency.

Data on the perceived psychological similarity defined by the PSPA test is shown in Table 2. The bigger the distance in the semantic space between a subject’s “Real Ego” and the other crew members, the bigger will be the perceived difference, and vice versa. During isolation, we detected statistically non-significant (according to the Wilcoxon t-criterion) tendency for the decrease in distances, which could be interpreted as the increase in the perceived similarity throughout their stay in the chambers. It is important to note that during the second testing in isolation (MD13), as well as after isolation, we detected statistically significant differences between “Ideal Ego” and perceived images of other subjects (*р* = 0.028).

**TABLE 2:** Average distances between the Real Ego of each crew member and the perceived images of other participants.

Perceived subject	Baseline	MD6	MD13	After isolation
A	2.66	3.38	2.5	1.87
B	3.11	3.71	2.67	2.81
C	2.68	2.4	4.01	2.76
D	4.07	4.56	3.48	3.62
E	2.48	2.41	2.49	2.49
F	3.18	2.84	2.43	1.73
Group average	3.03	3.22	2.93	2.55

**FIGURE 9:**
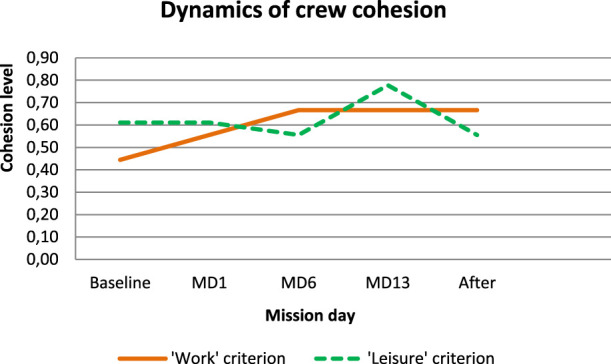
Dynamics of crew cohesion according to sociometry.

In general, the sociometric test, a study of group values and perceived images of the crew members, shows the increase in crew cohesion during isolation (Figure 9). Only one member of the group seemed to be not compatible with the rest.

### Effects of using various methods and means of psychological support on isolation and confinement of a small group

Results obtained with the KTS are shown in Table 3. It is evident that almost all subjects demonstrated evident dominance of judging. Three of them (A, D, and E) also gave preference to sensing and thinking, so they can be attributed to the sensory-planning type. Subject B, who, as we found out, did not share the crew’s common values, demonstrated dominance of intuition. Furthermore, we correlated the subjects’ types of processing information with their preferences to types of psychological support.

**TABLE 3:** Results of temperament testing using the KTS questionnaire.

Subject	Extraversion	Introversion	Sensation	Intuition	Thinking	Feeling	Judging	Perceiving
A	0	10	14	6	14	6	20	0
B	5	5	5	15	13	7	11	9
C	4	6	7	13	8	12	16	4
D	7	3	17	3	18	2	16	4
E	3	7	11	9	10	10	17	3
F	6	4	9	11	9	11	14	6

During the preparatory stage of the experiment, all subjects gained information about the opportunity to install their own video and audio content, including photos and books, on their personal tablet computers. This content could be used during their leisure time.

To provide medical safety criteria, individual intolerance to VR utilization as a means of psychological support was defined by the interview and special neurological tests in sitting and standing positions. Two subjects complained about symptoms of motion sickness (vertigo and nausea) in their common life. Expanded neurological examination defined that they had nystagmus after a VR session. No other neurological symptoms like tremor or instability during Romberg’s test were found. During the isolation period, these subjects did not complain about any vestibular problems during VR sessions.

According to the “psychological support” questionnaire, the average values of “PS from communication” were positively correlated both with the degree of association of the subjects with the crew (perception of themselves as part of the group) and with the level of support from the crew (*ρ* = 0.888, *p* < 0.05 and *ρ* = 0.858, *p* < 0.05, respectively). The “extraversion” parameter from KTS had a positive correlation with the indicator of psychological support from communication (i.e., social support) with the duty crew (*ρ* = 0.896; *p* < 0.05). At the same time, persons with a tendency for introversion preferred to use psychological support provided *via* virtual reality technology (*ρ* = −0.896, *p* < 0.05). This allows us to assume a direct relationship between the amount (volume) of communication in the crew and with the duty crews, a sense of unity (association) with the crew, and the favorable and supportive influence of this communication on the subjects with the dominance of extraversion.

In addition, it was shown that the indicator of the need for generalization and interpretation of information (KTS “thinking” scale) was negatively correlated with support from VR (*ρ* = −0.858, *p* < 0.05). Also, the need for the formal conservative pragmatic data and common sense (“sensation” scale, according to Keirsey) was positively correlated with the need for PS based on VR (*ρ* = 0.888, *p* < 0.05).

Not all the data from the video-recorded self-reports before and after VR sessions were available due to technical problems. The data describing basic emotions defined using Noldus FaceReader software *via* the subjects’ facial expressions showed that after VR sessions, the level of so-called neutral facial expression and «Anger» decreased, while the levels of «Happy», «Surprise», and «Arousal» increased. The detected parallel increase of «Disgust » contradicts the positive tendencies mentioned previously. Still, this result correlates with the previous data from the dry immersion study, where some subjects complained about the return of negative emotions immediately after leaving the virtual world and returning to the real stressful conditions (Rozanov et al., 2022). Also, similar to the dry immersion study, after VR sessions, we found a statistically significant decrease in cognitive mistakes (slips of the tongue and expletives) in self-reports (*T* = −2,101; *p* < 0.05). For subjects D, E, and F, after two sessions of VR-based PS, we also detected a statistically insignificant decrease in night awakenings and the increase in sleep duration.

## Discussion

The results obtained in 14 days of isolation did not completely confirm the first initial hypothesis. Data from questionnaires mostly contradict the concept about anxiety growth under isolation, crowding, and confinement. Only several negative changes were detected during the preparatory stage and the first day of isolation. These results confirmed numerous studies showing that novices mostly regard the period before the mission as more stressful than the space flight itself (Kozerenko et al., 2001).

At the same time, utilizing measurements not influenced by the subjective self-perception, we detected some negative changes. First of all, we found a decrease in sleep duration and increase in the number of awakenings in several subjects throughout the isolation period. Also, certain changes in crew facial expression during morning and evening DPСs testified that less positive feelings and more negative feelings like anger and sadness were expressed. However, the most negative emotional reactions in the middle of the mission were related not only to isolation itself but also to the particular events when a particular subject was directly involved in them. Also, some changes in the voice acoustic parameters were correlated with the anxiety level.

We expected that the lack of personal space and crowding could cause an increase in conflict tension as a result of territorial behavior. However, the data gained testified that crew cohesion increased under isolation and confinement which was expressed in the increase in the value similarity and decrease in psychological distances in the crew as well as in sociometric data. Although not knowing each other well enough before the study (less psychological similarity in the baseline), the subjects, in the course of isolation, increasingly perceived each other as psychologically similar.

This helped crew members obtain psychosocial support from each other, as it was previously detected in the much longer isolation studies of the international crews in MARS-520 and SIRIUS-19 (Šolcova et al., 2021a; Šolcova et al., 2021b). That answered the question why the ESKIS subjects did not report significant changes in their mood under stressful conditions. It corresponds to our second hypothesis that subjects with better communicative skills, extraversion, and proneness to the communication could withstand isolation, monotony, and confinement receiving support *via* contacts with their social surrounding (Kuznetsova et al., 2018). Another subgroup of more introverted subjects preferred to utilize VR with its more predictable structured content to successfully compensate sensory deprivation, monotony, and lack of personal space.

It should be noted that the subjects’ exposure to isolation and crowding was relatively short, and thus we were unable to observe the effects of longer isolation studies, such as increases in crew autonomy and psychological asthenia or tension (Gushin, 2021). There is a possibility that longer duration of crowding within an extremely confined space could be a significantly more detrimental factor for the subjects’ psychological state, cognitive functions, and social behavior, and it should be investigated in further studies with a similar design.

## Conclusion

In conclusion, despite the 14 days of isolation, monotony, and crowding, the mixed-gender crew of mostly inexperienced subjects successfully adapted to the stressful environment. Further studies are needed, but we consider that the most part of negative psychophysiological effects of isolation, monotony, and crowding was prevented, like in the extended space flights (Rozanov et al., 2021), by the effective utilization of psychological support. Also, the process of team building under the influence of numerous stressful factors played an important role in the successful adaptation of the group. It should be noted that the degree of need for various types of psychological support was associated with qualities of the subjects such as extroversion–introversion. In subjects with a tendency for extraversion, VR-based PS played a smaller role than established social ties with other group members and duty teams. In addition, the extroverted subjects had an increased need for support based on photographic materials and books, while the introverted subjects had an increasing need for VR-based PS.

## Data Availability

The raw data supporting the conclusion of this article will be made available by the authors, without undue reservation.
